# A multi-component cognitive behavioural intervention for the treatment of fear of falling after hip fracture (FIT-HIP): protocol of a randomised controlled trial

**DOI:** 10.1186/s12877-017-0465-9

**Published:** 2017-03-20

**Authors:** Maaike N. Scheffers-Barnhoorn, Jolanda C. M. van Haastregt, Jos M. G. A. Schols, Gertrudis I. J. M. Kempen, Romke van Balen, Jan H. M. Visschedijk, Wilbert B. van den Hout, Eve M. Dumas, Wilco P. Achterberg, Monica van Eijk

**Affiliations:** 1 Department of Public Health and Primary Care, Leiden University Medical Center, Postbox 9600, Leiden, 2300 RC The Netherlands; 2 Department of Health Services Research and Care and Public Health Research Institute (CAPHRI), Faculty of Health, Medicine and Life Sciences, Maastricht University, Maastricht, The Netherlands; 3 Geriatric Center and Nursing Home Antonius Binnenweg, Laurens, Rotterdam, The Netherlands; 4 Department of Medical Decision Making and Quality of Care, Leiden University Medical Center, Postbox 9600, Leiden, 2300 RC The Netherlands; 5 Medical Psychology department, The Tjongerschans Hospital, Postbox 10500, 8440 MA Heerenveen, The Netherlands

**Keywords:** Fear of falling, Hip fracture, Geriatric rehabilitation, Randomised controlled trial, Cognitive behavioural therapy

## Abstract

**Background:**

Hip fracture is a common injury in the geriatric population. Despite surgical repair and subsequent rehabilitation programmes, functional recovery is often limited, particularly in individuals with multi-morbidity. This leads to high care dependency and subsequent use of healthcare services. Fear of falling has a negative influence on recovery after hip fracture, due to avoidance of activity and subsequent restriction in mobility. Although fear of falling is highly prevalent after hip fracture, no structured treatment programme is currently available. This trial will evaluate whether targeted treatment of fear of falling in geriatric rehabilitation after hip fracture using a multi-component cognitive behavioural intervention (FIT-HIP), is feasible and (cost) effective in reducing fear of falling and associated activity restriction and thereby improves physical functioning.

**Methods/design:**

This multicentre cluster randomised controlled trial will be conducted among older patients with hip fracture and fear of falling who are admitted to a multidisciplinary inpatient geriatric rehabilitation programme in eleven post-acute geriatric rehabilitation units. Fifteen participants will be recruited from each site. Recruitment sites will be allocated by computer randomisation to either the control group, receiving usual care, or to the intervention group receiving the FIT-HIP intervention in addition to usual care. The FIT-HIP intervention is conducted by physiotherapists and will be embedded in usual care. It consists of various elements of cognitive behavioural therapy, including guided exposure to feared activities (that are avoided by the participants). Participants and outcome assessors are blinded to group allocation. Follow-up measurements will be performed at 3 and 6 months after discharge from geriatric rehabilitation. (Cost)-effectiveness and feasibility of the intervention will be evaluated. Primary outcome measures are fear of falling and mobility.

**Discussion:**

Targeted treatment of fear of falling may improve recovery and physical and social functioning after hip fracture, thereby offering benefits for patients and reducing healthcare costs. Results of this study will provide insight into whether fear of falling is modifiable in the (geriatric) rehabilitation after hip fracture and whether the intervention is feasible.

**Trial registration:**

Netherlands Trial Register: NTR 5695.

## Background

Global healthcare is challenged by an ageing population. The number of people aged ≥60 years is expected to increase from 900 million in 2015 up to 2 billion in 2050 worldwide (i.e. 12 and 22%, respectively, of the population). For the oldest old (aged ≥80 years), the calculated trend is an increase from 120 million in 2015 up to 434 million in 2050 [1]. Despite the diversity of experienced health in older age, many older adults often face numerous health conditions affecting their physical and mental capacity, independence, autonomy and overall well-being and quality of life. At present there is no evidence that the current generation of older adults is in better health in their older years compared with the previous generation [2]. Due to the relative increase of elderly in the global population, medical and formal care consumption is increasing, placing a burden on healthcare systems and caregivers worldwide. Therefore, healthcare strategies should be aimed at optimising the older adult’s functional ability and supporting their independence.

Falls and fall-related injuries, specifically hip fractures, are a major health problem for older adults, threatening physical and functional ability [3–5]. Annually 1.6 million older adults worldwide sustain a hip fracture and this number is expected to reach 4.5 million in 2050 [2]. A hip fracture in older adults is associated with poor functional outcome, with a 1-year mortality rate of approximately 30% [3, 4, 6, 7]. Despite surgery and subsequent rehabilitation programmes, many older hip fracture patients experience permanent functional disability as a result of the fracture, with only 40–60% recovering to their pre-fracture level of mobility within 1 year after fracture. 6 months after a fracture, about 42–71% have regained their pre-fracture level of functioning in basic activities in daily living (ADL) [3–5, 8]. Approximately 10–20% are unable to return to their prior residence [5]. The degree of disability may be even greater for frail older adults in need of extensive rehabilitation within an inpatient setting. Therefore, interventions aimed at optimising functional recovery after hip fracture and decreasing future fall risk are important to improve outcome for individual patients, and to reduce the burden on (in)formal care and therefore society.

Social demographic factors (age, gender), pre-fracture physical condition and functioning (walking ability, level of independence in ADL, co-morbidity, hand grip strength), psychological factors (cognitive functioning, depression, fear of falling), pain and anaemia influence functional outcome after hip fracture [4, 9–12]. However, only a few of these factors are potentially modifiable and thus eligible to be targeted in an intervention strategy to improve functional outcome. In this context, fear of falling is of specific interest as it has an even greater impact on recovery after hip fracture than does cognitive state, depressive symptoms, or level of perceived pain [11]. In addition, fear of falling is important as it is highly prevalent in both community-dwelling older adults (54%) [13, 14] and in patients who have sustained a hip fracture (50–65%) [15, 16].

Fear of falling is defined by Tinetti et al. as: ‘*a lasting concern about falling that leads to an individual avoiding activities that he/she remains capable of performing’* [17]. Consequences of fear of falling (and activity avoidance due to fear of falling) are increased risk of falls, decreased mobility/balance performance, loss of independence, lower social participation, and lower health-related quality of life [13, 18]. Therefore, it not only affects physical functioning, but also psychosocial functioning. Specifically, after a hip fracture, fear of falling is associated with a reduction in time spent on exercise during rehabilitation [15] which, in turn, impedes functional performance.

In the Netherlands, about 25–30% of elderly hip fracture patients receive inpatient multidisciplinary rehabilitation care following surgery, due to the acute decrease in their physical functioning and associated dependency in ADL. This vulnerable patient group is discharged from hospital to ‘geriatric rehabilitation’ (GR), a multidisciplinary inpatient rehabilitation programme within post-acute GR units in nursing homes. The rehabilitation programme, which is led by an elderly care physician, includes physical - and occupational therapy, and treatment of comorbidities. In GR, fear of falling is highly prevalent among patients with hip fracture (63%) [16].

Targeted treatment of fear of falling during rehabilitation after hip fracture could lead to reduction of fear of falling and the associated activity restriction and, therefore, to improved mobilisation, functional recovery and a higher level of independence. To our knowledge, no treatment programmes are currently available for the treatment of fear of falling among this specific patient population [15, 19]. However, several programmes are available for the treatment of fear of falling for community-dwelling older adults. For example, the Netherlands has an adapted Dutch version of ‘A Matter of Balance’ [20, 21]. This multicomponent cognitive behavioural group programme has proven cost-effective in treating fear of falling and has been implemented nationally [22–24]. Recently a home-based version of ‘A Matter of Balance’ was developed and this latter programme also proved (cost)effective in reducing fear of falling and associated activity restriction, disability and indoor falls [25, 26].

Partially based on the Dutch version of ‘A Matter of Balance’, and specifically developed for the multidisciplinary GR setting, the multi-component cognitive behavioural FIT-HIP intervention has been developed. It is directed at reducing fear of falling and the associated avoidance of activities and increasing self-efficacy and daily functioning among hip fracture patients admitted to GR. This multicentre cluster randomised controlled trial (RCT) will examine whether the FIT-HIP intervention is feasible and (cost)effective in reducing fear of falling and, therefore, improving functional outcome in hip fracture patients in GR. In addition, it will assess whether the intervention is feasible for patients and healthcare professionals.

### Primary objective

In hip fracture patients admitted to multidisciplinary inpatient GR, to compare the effect of the FIT-HIP intervention with usual care in GR, with respect to reducing fear of falling (measured with the Falls Efficacy Scale-International) and improving gait and balance (measured with the Performance-Oriented Mobility Assessment).

### Secondary objectives

- To compare the effect of the FIT-HIP intervention with usual care with respect to improving the degree of independence in ADL (Barthel index), ambulation ability (Functional Ambulation Categories) and walking speed.
- To compare the number of fall incidents, mortality, hospital (re)admission and psychosocial functioning (social participation after discharge from GR, measured by the Utrecht Scale for Evaluation of Rehabilitation-subscale Participation; and quality of life, measured by the EuroQol 5D) between the FIT-HIP intervention and usual care.
- To examine the feasibility of the FIT-HIP intervention for participants and therapists conducting the FIT-HIP intervention.
- To perform an economic evaluation, consisting of a cost analysis and cost-utility analysis, comparing the FIT-HIP intervention with usual care. Costs will be measured from a healthcare perspective.

## Methods/design

### Study design

**Fig. 1:**
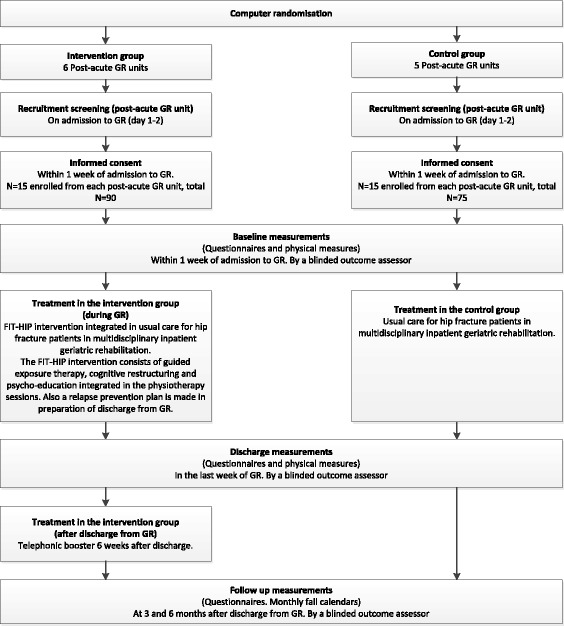
Procedures of the FIT-HIP clustered randomised controlled trial. GR = geriatric rehabilitation (multidisciplinary inpatient rehabilitation programme)

This multicentre cluster RCT will be conducted among 165 patients with hip fracture and fear of falling, who are admitted to a multidisciplinary inpatient GR programme in post-acute GR units in Dutch nursing homes. For these hip fracture patients in GR, this RCT compares usual care (control group) with an intervention group that includes the addition of the FIT-HIP intervention to the usual care. The FIT-HIP intervention is aimed at reducing fear of falling. Figure 1 presents an overview of the study design. Simultaneously, a process evaluation will be performed to assess the feasibility of the programme.

This study protocol was approved by the Ethics Committee of the Leiden University Medical Center (9 September 2015; P15.212). In addition, the Board of Directors and (if applicable) the research committees of the participating recruitment sites (post-acute GR units of nursing homes) provided consent to participate in the FIT-HIP intervention study.

Prior to baseline assessments and to starting the FIT-HIP treatment (in the intervention group), written consent will be obtained from participants.

### Setting

The department of Public Health and Primary Care (PHEG) of the Leiden University Medical Center will coordinate the FIT-HIP study. Eleven post-acute GR units from nursing homes in the province South Holland are included in this study, most of which work in close collaboration with the PHEG through the University Network for the Care-sector South Holland (UNC-ZH). Annually, the eligible post-acute GR units each have ≥50 patients admitted for GR after orthopaedic events (e.g. trauma, elective surgery or amputation).

### Participants (and eligibility criteria)

Study participants are patients aged ≥65 years, admitted to one of the 11 participating post-acute GR units for a geriatric rehabilitation programme following surgical repair of a hip fracture, and concerned to fall. Fear of falling is assessed within the first week of admission to GR, using the 1-item fear of falling question (‘*Are you concerned to fall?*’). This question has five answer categories (*never – almost never – sometimes – often – very often*). Patients are eligible to participate if they answer this question with *‘sometimes, often, or very often’*

An exclusion criterion for this trial is any condition interfering with learnability, e.g. a diagnosis of dementia, major psychiatric disease, or a score of > 1 on the Hetero-anamnesis List Cognition (HAC) [27]. The HAC is derived from the Mini Mental State Examination (MMSE) and is used to explore the presence of premorbid cognitive disabilities. A relative/informal caregiver is asked if there were problems concerning orientation, language, memory, planning and execution of activities, and to which degree the patient needed assistance or professional therapy for these problems. A score of > 1 is suggestive for premorbid cognitive problems. Other exclusion criteria for this trial are a limited life expectancy (<3 months), the presence of a pathological hip fracture, a pre-fracture Barthel index score of < 15, and insufficient mastery of the Dutch language.

### Randomisation (and allocation)

Of the 11 post-acute GR units, six will be randomly allocated by computer-generated randomisation to conduct the FIT-HIP intervention and five are allocated to the control group (usual care). Hip fracture patients will be screened for eligibility for the FIT-HIP study on admission to these post-acute GR units. For this trial, each post-acute GR unit will include a maximum of 15 participants (in order of succession in which patients are admitted to GR, eligible, and willing to participate). Participants will receive treatment (usual care, or the addition of FIT-HIP intervention to usual care) according to the randomisation of the post-acute GR unit to which they are admitted.

### Usual care (control group)

Usual care consists of an inpatient multidisciplinary rehabilitation programme (GR) for patients with a hip fracture. This rehabilitation programme is led by an elderly care physician. It comprises physical therapy sessions focussing on balance and gait exercises, and improving muscle strength. The nursing staff and an occupational therapist are also involved in coaching patients in performing ADL, e.g. going to the toilet, and self-care. Each participating post-acute GR unit employs a care-pathway GR, containing formalised agreements on the contents of the multidisciplinary rehabilitation process, such as therapy intensity and assessments during rehabilitation. In general, a patient will receive 5-6 sessions of physiotherapy per week.

### The FIT-HIP intervention

The FIT-HIP intervention is a multi-component cognitive behavioural intervention aimed at reducing fear of falling in hip fracture patients in GR. It is an individualised treatment programme, tailored to the individual needs, preferences and capacities of the participant. It is coordinated and primarily conducted by physiotherapists. The programme is combined with regular exercise training during the physiotherapy sessions in GR (usual care). The physiotherapists are part of the multidisciplinary GR healthcare team of the participating post-acute GR unit and have experience in the field of (orthopaedic) rehabilitation of frail older adults. Prior to participant recruitment, two physiotherapists per intervention post-acute GR unit will be trained to conduct the FIT-HIP intervention. Also, for each intervention post-acute GR unit, one psychologist (who is part of healthcare team concerned), will be briefed on the intervention and will participate in part of the training.

The psychologists are trained to function as a coach for the physiotherapists, assisting them with cognitive restructuring when they need advice on this subject. If required, they also assist in the additional treatment of participants, e.g. for more complex psychiatric problems such as generalised anxiety disorder or post-traumatic stress disorder (in the event that this only became apparent during admission and could not have been considered an exclusion criterion).

All elements of the FIT-HIP intervention are described in more detail below. The guided exposure to mobility- related activities is the core element of the intervention and is also applied by the nursing staff in the process of mobilisation during GR. The nursing staff was trained in the concepts of guided exposure and instructed how to administer this. The treatment plan for the mobilisation process (guided exposure) is made by the physiotherapists. Based on the existing communication procedures for each post-acute GR unit, communication protocols will be drafted on how the physiotherapists keep the nursing staff updated on the current status of treatment plans for the individual participants.

#### Guided exposure

Guided exposure to the situations that participants fear is the core element of the FIT-HIP intervention. In the case of fear of falling, the feared situation will be a form of activity and therefore the exposure to that situation will be practical training of an activity. These fearful situations are assessed for each patient individually during the intake to GR. In rehabilitation after hip fracture the feared situations may be basic (but fundamental) for the mobilisation process and performing ADL. Examples of assessed situations are: standing, transfer (from bed to chair and vice versa), toilet use, walking inside/outside, and staircase walking. In the intervention, it is also important to focus on participation activities. Therefore, the physiotherapist also assesses which (more complex) activities in daily living the participant considers important or desirable to able to perform, and which of these may lead to fear of falling, e.g. cycling or using public transport.

**Fig. 2:**
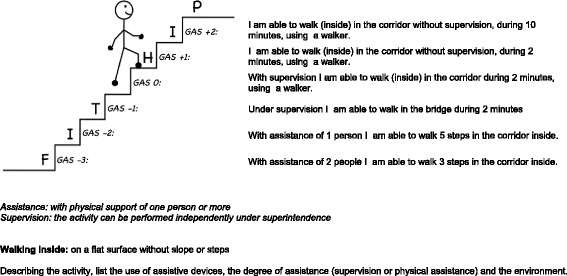
Example of a FIT-HIP fear ladder (*walking inside*)

For each of these feared situations, guided exposure will be conducted by means of a separate fear hierarchy. In the FIT-HIP intervention the fear hierarchy is represented in a ‘fear ladder’. Each ‘fear ladder’ contains six steps, each step representing a goal. Goals for exposure are ranked according to the intensity of fear of falling it gives rise to, and edited in such a manner that there is an increasing intensity of concern/fear. Goals are formulated in accordance with the Goal Attainment Scaling (GAS) method [28, 29]. The GAS is a technique for developing individualised, scaled descriptions of treatment goals. It is a method to evaluate the (rehabilitation) therapy. Goals are formulated in a SMART manner (specific, measurable, acceptable, realistic and defined in time), in collaboration with the patient in order to relate to the personal interests and social environment of the patient. The goals are scaled from −3 to +2, with −3 being deterioration in function, −2 the starting point (current situation when starting the therapy) and 0 being the primary goal. At −1 there is improvement in function but the primary goal in not yet achieved, and at +1 and +2 the function is better than the primary goal. All treatment goals are formulated as functional goals of improvement of mobility. They are not formulated as goals to (primarily) decrease fear. The fear ladders are evaluated with the participant every week and adjusted if necessary. Figure 2 is an example of a FIT-HIP fear ladder.

The fear ladders are incorporated in the individual FIT-HIP therapy plan. This therapy plan forms a guiding principle for applying the guided exposure in the process of mobilisation. The exposure takes place gradually, with increasing intensity, in a predictable and controllable manner, and under supervision of the physiotherapist. Due to this repeated graded exposure to the feared situation, the fear is expected to initially increase in the presence of the physiotherapist, but to lessen and gradually fade out during the experience of the activity. Guided exposure will be performed during each physiotherapy session during GR (combined with other physical exercises, such as strength/balance). Participants are also encouraged to practise exposure outside of the therapy sessions (homework). The nursing staff will have a supporting function in this process. The nursing staff is regularly briefed by the physiotherapist to engage in the current principles of the guided exposure for the individual patient.

#### Cognitive restructuring

This is based on the principles of cognitive behavioural therapy whereby the combination of applied behaviour and effectively recognising and managing negative/unrealistic thoughts and learning to apply realistic thoughts are the key components. Physiotherapists are trained to apply these principles during the therapy sessions. Also, at least once during the rehabilitation, a worksheet is filled in to structure this process (describing the event, thoughts, feeling, behaviour, consequence) and helping the participant to formulate realistic thoughts. The patient learns to examine his/her thoughts and beliefs, and the effect this has on behaviour and feeling (anxiety). This principle is also incorporated in the relapse prevention plan.

#### Psycho-education

During the initial phase of rehabilitation, shortly after admission to GR, information is given to the participant on anxiety, fear of falling, consequences of fear of falling and self-help possibilities. The rationale and background of guided exposure will be explained. Also, the influence of thoughts/beliefs on emotion and behaviour will be explained (background of the cognitive restructuring).

In the final phase of rehabilitation, when a patient is in preparation of discharge (home), the psycho-education will focus on home safety. This will be processed in the relapse prevention plan.

#### Relapse prevention plan

In preparation of discharge from GR to the home situation, a relapse prevention plan for fear of falling will be made. The purpose of this plan is to assess situations/circumstances (in the home situation) in which the patient is at risk of a relapse. By means of this plan, the physiotherapist prepares the participant to anticipate these situations and to prevent falling back into old habits in potential fearful situations.

The relapse prevention plan will be worked out and given to the patient as a ‘Staying Active Plan’. It consists of three elements: 1) General home safety and fall prevention; 2) Individual advice for safe ambulation and staying active. Individual advice for use of walking aids/assistance is given, with precautions if necessary. Also, two individualised physical exercises are described that are recommended for the patient to stay active and in condition in the home situation. Also, if necessary with precautions. The therapist will also discuss that it can be useful to have a buddy to do these exercises with, and who that may be for the patient; 3 (Preventing) a relapse. Information is given about preventing and recognising a relapse, and advice as to what is helpful when a relapse occurs.

#### Telephonic booster

Six weeks after discharge from GR the physiotherapist conducts a telephonic booster intervention. The purpose of this booster is to evaluate the fear of falling in the first weeks after discharge, discuss difficulties concerning fear of falling and activity restriction, discuss the use of the relapse prevention plan and, if necessary, give new advice for dealing with or preventing fear of falling.

#### Motivational interviewing

Physiotherapists will also be trained to use motivational interviewing techniques for the guidance of their patients. Motivational interviewing is a client-centred, goal-oriented counselling technique that is used to explore and reinforce the patient’s internal motivation for behavioural change. By exploring and resolving ambivalence, it aims at evoking behavioural change [30]. In the FIT-HIP intervention, the motivational interviewing techniques can assist the physiotherapist to explore which (rehabilitation) goals are important for the individual participant, in order to personalise the treatment goals.

### Duration of the FIT-HIP intervention

The FIT-HIP intervention, integrated in the usual care, will be conducted during the entire period that the participant is admitted to GR. The duration of the inpatient GR is determined for each participant individually, and is therefore variable. On average, the duration of admission to GR for rehabilitation after hip fracture is 6 weeks. During the trial, the following is registered: i) total duration of GR in days, ii) number of therapy sessions during GR, iii) duration of therapy sessions, and iv) (in the intervention group) performance of the individual components of the FIT-HIP intervention; all these elements can be used as confounding variables in the final outcome analyses.

### Blinding

Both the participants and the independent research assistants assessing the outcome measurements are blinded to the group allocation. They are not aware of what usual care is/should be and what the addition of the FIT-HIP intervention is. Healthcare professionals working at the recruitment sites are aware of the allocation status, as the intervention group has been specifically trained to perform the intervention. They are instructed not to inform the participants, family members and the research assistants assessing outcome measures about the allocation status. The main researcher (MSB) was involved in providing the training for the intervention and therefore cannot be blinded in the initial phase of this trial. For data analysis, the database will be processed to blind data to the initial allocation.

To warrant the blinding of participants in the control group (who receive usual care with possibly no specific treatment for or notice of the fear of falling) a dummy intervention is given in both the control and intervention group. The dummy intervention is an information brochure containing information about fear of falling, its consequences, and possibilities for seeking medical attention or help for this problem. This is regarded as an appropriate dummy intervention, as healthcare strategies directed at reducing risk of falling in older adults that use educational interventions alone, have not proven effective [31]. Therefore, we do not expect this information brochure to have a significant effect on the fear of falling.

### Effect evaluation

#### Primary outcome

1. Mean difference in the Tinetti Performance Oriented Mobility Assessment (POMA) score [32, 33] at discharge from GR (or at a maximum of 3 months after admittance to GR), compared between FIT-HIP intervention and usual care. The POMA is a measure of mobility function (gait and balance).

2. Mean difference in the Falls Efficacy Scale International (FES-I) score [34–36] at discharge from GR (or at a maximum of 3 months after admittance to GR), compared between FIT-HIP intervention and usual care. The FES-I is a measure of fear of falling.

#### Secondary outcomes

Table 1 gives an overview of the secondary outcome measures in the effect evaluation. For these outcome measures, at discharge from GR, mean differences between the intervention and control group will be assessed.

**Table 1:** Secondary outcome measures in the FIT-HIP trial

Domain	Assessment	Description	Time point(s)
Physical functioning and activity	Barthel index [40]	Activities in daily living. Measures (in)dependence in personal care (eating, dressing, bathing, going to the toilet) and mobility.	BA, DA, FU1, FU2
	Functional ambulation categories [41]	Evaluates ambulation ability, describing the degree of human support the person needs when walking.	BA, DA, FU1, FU2
	10 meter walk test [42, 43]	Assesses walking function/speed.	BA, DA
	Activity restriction due to fear of falling	Assessed in questionnaire, asking if participant has experienced restriction of activity due to the fear of falling.	BA, DA, FU1, FU2
Falls, complications and health care service usage	Falls (and fall-related injury)	Number of fall incidents and medical attention required as a result of the fall. Assessed using monthly fall calendars.	BA, DA, FU1, FU2
	Complications during GR	Number and type of complication occurring during GR. Assessed by elderly care physician (ECP) in monthly calendars.	Until discharge from GR
	Hospital (re)admission	Number of hospital readmissions and days in hospital. Assessed by ECP in monthly calendars during GR and questionnaire at discharge from GR. Assessed by participants using questionnaire in FU.	DA, FU1, FU2
	Mortality		DA, FU1, FU2
	Healthcare consumption after discharge	Number of contacts with health and community services. Assessed by participants using questionnaire in FU.	FU1, FU2
Other outcome characteristics of GR	Duration of admission to GR	Number of days admitted to GR. Assessed by ECP (questionnaire).	DA
	Total amount of therapy in GR	Number of hours of physiotherapy and of contact with ECP. Assessed by physiotherapists in weekly calendars and by ECP in monthly calendars.	Until discharge from GR
	Discharge location	Location of the residence to which participant is discharged after GR. Assessed by ECP (questionnaire).	DA
Health-related quality of life	EuroQol 5D (EQ5D) [44]	The three-level EuroQol 5D (EQ-5D) is a standardised instrument for measuring generic health status. It can be used for calculating quality adjusted life years (QALYs), for the economic evaluation.	BA, DA, FU1, FU2
Participation	The Utrecht Scale for the Evaluation of Rehabilitation-Participation. (USER-P) [45]	Assesses (limitations in) participation in relation to health problems.	BA, FU1, FU2

*BA* baseline assessment (pre-intervention), *DA* discharge assessment (post-intervention), *FU1* follow-up 1 assessments, 3 months after discharge from GR, *FU2* follow-up 2 assessments, 6 months after discharge from GR, *ECP* elderly care physician, *GR* geriatric rehabilitation (multi-disciplinary inpatient rehabilitation programme), *EQ-5D* EuroQol 5D, *USER-P* Utrecht Scale for the Evaluation of rehabilitation - participation

#### Additional variables

Table 2 gives an overview of the additional variables assessed in this trial.

**Table 2:** Additional variables assessed in the FIT-HIP trial

Domain	Assessment	Description	Time point(s)
Socio-demographics	Age, gender, marital status, type of residence prior to hip fracture		BA
General health and physical functioning	Functional comorbidity index (weighed) [46]	Assesses 18 comorbid conditions and their effect on physical functioning.	BA
	Medication use	Number and type of medication used by participants. Assessed by ECP (questionnaire).	BA, DA
	Assistive walking device	Type of assistive walking aid, used for indoor and outdoor usage. Assessed by questionnaire.	BA
	Use of formal care (home care) and informal care (given by relatives/volunteers)	Assessed by questionnaire.	BA, FU1, FU2
	Previous fall frequency	Number of falls in 6 months prior to hip fracture.	BA
	Handheld grip strength	Evaluated with dynamometer.	BA
	Nutritional status: Body Mass Index	Calculated by dividing bodyweight in kilograms by length in meters squared.	BA, DA
	Numeric Pain Rating Scale (NPRS) [47]	Assesses intensity of pain on an 11-point scale (0 –10).	BA, DA, FU1, FU2
Hip fracture (related) characteristics	Type of fracture, operation, weight-bearing capacity	Assessed by ECP (questionnaire).	BA
	Duration of hospital admission due to hip fracture	Number of days in hospital.	BA
	Complications during hospital admission due to hip fracture	Number and type of complications. Assessed by ECP (questionnaire).	BA
Neuropsychological factors	Mini Mental State Examination (MMSE) [48, 49]	Screens for cognitive disorders and dementia	BA
	Geriatric Depression Scale, 8-item (GDS-8) [50]	Short adapted version of the GDS-30. Developed to screen depression in nursing home population.	BA
	Hospital anxiety and depression scale – subscale anxiety (HADS-A) [51]	Screens for anxiety.	BA
	Utrecht Coping List; subscales active and passive coping. (UCL) [52]	Assesses coping mechanism. Questionnaire assesses how a person deals with problematic situations in general.	BA
	Pittsburgh Rehabilitation Participation Scale [53]	Participation/motivation for physiotherapy (PT) during GR.	During every session of PT until discharge

*BA* baseline assessment (pre-intervention), *DA* discharge assessment (post-intervention), *FU1* follow-up 1 assessment, 3 months after discharge from GR, *FU2* follow-up 2 assessment, 6 months after discharge from GR, *ECP* elderly care physician, *NPRS* numeric pain rating scale, *MMSE* mini mental state examination, *GDS-8* geriatric depression scale, 8-item, *HADS-A* hospital anxiety and depression scale – subscale anxiety, *UCL* Utrecht’s coping list, *PT* physiotherapy

### Process evaluation

To determine the feasibility of the FIT-HIP intervention, a process evaluation will be conducted in accordance with the theory of Saunders et al. [37] Using a mixed-method approach, information about reach, fidelity, exposure, satisfaction and barriers for applying the programme will be assessed. Table 3 gives an overview of the measurement instruments used to collect these data.

**Table 3:** Outcome measures of the FIT-HIP process evaluation

Component and definition	Operationalisation	Measurement instruments									
		SLog	QpD	QpF1	QpF2	Ip	It	Qt	BLog	Sq	D
Reach											
Proportion of the intended target population that participated in the programme	Refusal and dropout rate. Reasons for withdrawal									+	+
Fidelity											
Extent to which the elements of the intervention were implemented as planned	Per therapy session: registration of which intervention components were performed	+									
	Per therapy session: reasons for deviation from individual FIT-HIP therapy plan	+									
	Reasons for deviation from protocol						+				
Dose received - Exposure											
Extent of participants’ active engagement in and receptiveness to the programme	Per therapy session: extent of active engagement in therapy	+									
	In general: use of relapse prevention plan (Staying Active Plan)			+	+				+		
Dose received - Satisfaction											
Satisfaction of participants and therapists with the programme	Overall opinion about the intervention		+	+	+	+	+	+			
	Opinion about the value of the intervention		+	+	+	+	+	+			
	Opinion about the value of the main elements of the intervention		+	+	+	+	+				
	Experienced burden		+			+	+				
Barriers											
The extent to which problems were encountered while applying the programme	Barriers in applying the (individual components of the) intervention.						+				
	Suggestions for improvement		+	+	+	+	+	+			
	Recruitment procedures						+				+
	Maintaining participant engagement						+				+

*SLog* physiotherapist session log, *QpD* evaluation questionnaire filled in by participant at discharge from GR, *QpF1* evaluation questionnaire filled in by participant at follow-up 1 (3 months after discharge from GR), *QpF2* evaluation questionnaire filled in by participant at follow-up 2 (6 months after discharge from GR), *Ip* Interview with participant, *It* interview with physiotherapist and psychologist, *Qt* evaluation questionnaire filled in by GR team members: elderly care physician, nursing staff and psychologist, *BLog* booster log, registration of telephonic booster, *Sq* screening questionnaire filled in at admission to GR, *D* data recorded by research assistants during study period

#### Therapist data

In the intervention arm of this trial, physiotherapists will register per session which elements of the intervention were conducted, reasons for deviating from the individual FIT-HIP therapy plan and the duration of the therapy sessions, using weekly calendars as session logs. Also, for each therapy session, the Pittsburgh Rehabilitation Participation Scale is filled in as a measure of the extent of active engagement of the participant in the therapy. At the end of the study, the physiotherapists and psychologists conducting the intervention will be invited to take part in qualitative group interviews to discuss in detail their satisfaction with the (components of the) intervention, experienced barriers applying the intervention and suggestions for improvement. Also, matters concerning participant recruitment and maintaining participant engagement will be discussed.

Other members of the GR team (the elderly physician and nursing staff) will be approached to fill in a short evaluation questionnaire about their general opinion of the intervention and to assess to what extent the individual FIT-HIP therapy plans were routinely discussed in the GR team.

#### Participant data

All participants in the intervention arm of this trial will receive evaluation questionnaires at discharge from GR and at follow-up (3 and 6 months after discharge from GR). In these questionnaires, information on experienced benefits and burden of the intervention, and suggestions for improvement of the intervention, will be assessed. In addition, qualitative interviews will be held with a (random) subgroup of the participants, to gain more insight into these matters.

### Economic evaluation

The economic evaluation consists of a cost analysis and a cost-utility analysis, both with a 6-month time horizon after discharge from GR. Costs will be measured from a healthcare perspective. In the cost-utility analysis, the difference in healthcare costs between the strategies will be compared to the difference in Quality-Adjusted Life Years (QALYs, calculated using the 3-level Dutch EQ-5D tariff [38] and the visual analogue scale for health). Estimated healthcare costs will include the costs of the FIT-HIP intervention (estimated from the study registration) and other healthcare utilisation (estimated using quarterly questionnaires filled in by the patients). Other healthcare utilisation will include care provided by general practitioners, consultations of medical specialists and paramedics, home care, informal care, hospitalisation, and residential care. A cost-price analysis will be performed for the FIT-HIP intervention; other healthcare items will be valued using standard prices.

### Sample size

This study tests the null hypothesis that there is no difference in POMA score between the intervention and control group at discharge from GR. The criterion for significance (alpha) was set at 0.050. The test is 2-tailed, which means that an effect in either direction will be interpreted. With a sample size of 40 in both groups, the study will have power of 80% to yield a statistically significant result. Based on our previous research, the minimal clinical relevant difference (mean difference of the POMA at discharge measurement) was set at -3.8, with the common within-group standard deviation at 6.0. The corresponding means are 17.0 vs. 20.8. This effect was selected as the smallest effect that would be important to detect, in the sense that any smaller effect would not be of clinical or substantive significance. It is also assumed that this effect size is reasonable, in the sense that an effect of this magnitude could be anticipated in this field of research.

Compensation for design effect and possible loss to follow-up was taken into account in the choice of sample size. For the design effect (cluster randomisation), the intraclass correlation coefficient (ICC) for the outcome measure POMA is expected to be 0.05 because of clustering of data and because there may be inequality of the numbers within clusters. For the possible loss to follow-up, specifically death in the 3-month rehabilitation phase is not expected be ≥10%. Instead of the 40 patients calculated with the power analysis, we will include 75 patients per group.

As 11 post-acute GR units were interested in participating, we decided to include one additional intervention post-acute GR unit, in case of unsuspected drop-out of one intervention location. Thus, we aim to include a total of 165 participants.

### Data analyses

Differences between the intervention and control group in characteristics of participants at baseline will be tested with chi-square tests for categorical variables, Mann-Whitney *U*-test for continuous variables with skewed distributions, and one-way ANOVA for normally distributed continuous variables. Given the hierarchical data structure, multilevel analyses will be used for continuous outcomes, and logistic Generalized Estimated Equation (GEE) analyses for dichotomous outcomes. Logistic GEE is preferred to logistic multilevel analyses because of the instability of the latter. Analyses will be based on an intention-to-treat principle and the level of significance will be set at *p* < 0.05. Missing data will be handled as missing (no imputation). Multilevel analyses will be performed with MLwiN. All other analyses will be performed with IBM SPSS statistics.

With regard to the qualitative data (assessed for the process evaluation), these will be analysed by means of coding techniques based on transcriptions of the qualitative interviews. In the economic evaluation, group averages will be compared using unequal-variance t-tests, according to the intention-to-treat principle. Costs will be compared to QALYs using net-benefit analysis. Multiple imputation will be used to account for missing values. Sensitivity analysis will be performed on the time horizon (base case 6 months vs. 12 months) and the utility measure (base case Dutch EQ-5D tariff vs. visual analogue scale for health).

## Discussion

At present, the functional recovery after a hip fracture in frail older adults is limited, resulting in a considerable amount of long-term disability. Therefore, a hip fracture has major consequences for individual patients, as well as for society, due to the costs of healthcare and the burden on caregivers. Based on the current literature, only a few factors influencing functional recovery after hip fracture could prove to be modifiable. As fear of falling is highly prevalent in hip fracture patients and leads to avoidance of activity, it is probably a significant factor contributing to limited recovery after hip fracture. To our knowledge this is the first RCT to evaluate the effect of treatment of fear of falling in this population. This multicentre cluster RCT will provide insight into whether targeted treatment of fear of falling during geriatric rehabilitation after hip fracture, using the FIT-HIP intervention, is effective in reducing fear of falling and associated avoidance of activities and, therefore, improving functional outcome after hip fracture.

The key component in this trial, guided exposure, is based on the principles of cognitive behavioural therapy. It encourages the systematic confrontation of feared stimuli (situations), in a graded approach. It is the preferred treatment in various types of anxiety disorders, including phobias. In the FIT-HIP programme, the guided exposure is used in conjunction with psycho-education and cognitive restructuring. The programme has been developed together with experts that developed a treatment programme on fear of falling in community-dwelling older adults, which was shown to effectively reduce the fear of falling [21–26].

Because the FIT-HIP programme is integrated in usual care, the additional costs are expected to be limited. In an earlier phase we conducted a small pilot study, aimed at testing the FIT-HIP training and the feasibility of the intervention for healthcare professionals and participants. The additional time spent on therapy for the purpose of this intervention appeared to be limited in the pilot, but will become clear after the evaluation of the intervention. Also, guided exposure was easily integrated in the usual care. Although the principles of guided exposure are often practiced in usual care, they are not generally as structured and intentional as in this intervention.

A strength of this study is that the feasibility for healthcare professionals and patients will be evaluated through a process evaluation. Cost effectiveness will also be assessed. If this intervention proves to be (cost)effective in improving functional outcome after hip fracture and is feasible, it could offer major benefits for individual patients, their (family) caregivers and for society.

This study also has some challenges. Cluster randomisation was chosen as the study design, as the risk of contamination of the FIT-HIP intervention on usual care would be too substantial in view of the complex nature of the intervention. All participating recruitment sites (post-acute GR units) employ a standardised care pathway for patients with hip fracture. This care pathway contains formalised agreements on the content of the multidisciplinary rehabilitation process [39]. As the post-acute GR units are all part of different Dutch care organisations, there could be subtle differences in the usual care for hip fracture patients. These differences (quantity and quality of the received therapy) will be assessed in the process evaluation.

A second challenge in this study, is the blinding. As the FIT-HIP intervention is compared to ‘care as usual’, blinding is only partially possible. Generally, participants should not be aware of what usual care is and what the addition of the FIT-HIP intervention could be. If, however, the usual care does not take note of the fear of falling, the participant could suspect being allocated to the control group. To limit this effect, all participants receive an information brochure on fear of falling and self-help possibilities. Educational interventions alone, aimed at increasing knowledge about fall prevention, have not proven to be effective in fall prevention and we therefore do not expect that this will contaminate the effect of the intervention [31]. The healthcare professionals (physiotherapists, psychologist and nursing staff) receive specific training for conducting the FIT-HIP treatment and are therefore aware of allocation; however, they are instructed not to inform the participants, family or research assistants. Outcome assessors (research assistants) are blinded to allocation.

In conclusion, this study will provide insight into whether fear of falling is modifiable in the rehabilitation process after hip fracture. The results of this trial will be disseminated in peer-reviewed journals and via professional and scientific conferences.

## Abbreviations

ADL: Activities of daily living
ECP: Elderly care physician
EQ-5D: EuroQol 5D
FES-I: Falls efficacy scale-international
FIT-HIP trial: Fear of falling intervention in hip fracture geriatric rehabilitation
GAS: Goal attainment Scaling
GDS: Geriatric depression scale
GR: Geriatric rehabilitation (multidisciplinary inpatient rehabilitation programme)
HAC: Hetero-anamnesis list cognition
HADS-A: Hospital anxiety and depression scale – subscale anxiety
LUMC: Leiden University Medical Center
MMSE: Mini mental state examination
NPRS: Numeric pain rating scale
PHEG: Department of public health and primary care
POMA: The tinetti performance oriented mobility assessment
PT: Physiotherapy
QALY: Quality-adjusted life years
RCT: Randomised controlled trial
UCL: Utrecht’s coping list
UNC-ZH: University Network for the Care-sector South Holland
USER-P: Utrecht Scale for the Evaluation of Rehabilitation-Participation.
